# Using video games to understand sex differences in attentional biases for weapons

**DOI:** 10.1371/journal.pone.0279360

**Published:** 2022-12-22

**Authors:** Gemma van Heyst, Myoungju Shin, Danielle Sulikowski

**Affiliations:** 1 School of Psychology, Charles Sturt University, Bathurst, Australia; 2 Perception and Performance Research Group, School of Psychology, Charles Sturt University, Bathurst, Australia; Albanian University, ALBANIA

## Abstract

Attentional biases for threatening stimuli of various kinds have been repeatedly demonstrated. More recently, sex differences in the strength of visual biases for weapons have been observed, with men exhibiting stronger biases than do women. In the current study we further explored this sex difference, by examining how immediate vicarious experience with weapons (via playing a violent video game compared to playing a non-violent video game) affected the visual attention for weapons. We found that the basic visual bias for weapons compared to non-weapons was replicated, as was the sex difference in the strength of this bias. We also observed that the context produced by playing a violent video game prior to the visual search task, produced some sex differences in responding that were not present after playing the nonviolent video game, providing modest evidence that men may be more prone to cognitive behavioural effects of violent video game play. Interestingly, there was some evidence that both sexes de-prioritised non-weapons during search after playing the violent, relative to the non-violent, video game. We recommend that future studies investigate the task dynamics that may have led to this effect.

## Introduction

Robust biases preferentially direct visual attention toward threatening stimuli, including venomous creatures, such as snakes and spiders [1–4], angry (male) faces [5], large predatory animals [6], and weapons [7–9]. The functional significance of such biases is presumed to lie in the benefits afforded by rapid detection of, and thus responses to, immediate threats in the proximate environment [3, 10, 11]. There is some evidence that a rudimentary visual sensitivity to snakes can manifest in individuals completely naïve to the danger snakes present (including human infants aged 8-14months, [12], and captive reared Japanese macaques, [13]. There is also, however, substantial evidence that experience influences the development of attentional biases, for example, vaccinated children exhibit attentional biases towards syringes, but not towards knives or pens [14]. The existence of a robust attentional bias for weapons [7, 9, 15] necessarily invokes experiential explanations.

The role of life experience in the development of attentional biases is especially salient with respect to the bias for weapons. Children engage in gender-congruent object preference from as young as 9 months of age [16, 17]. Due to early developing perceptual preferences [16, 18], and parenting influences [19] boys are much more likely to own, choose to play with, and spend a greater amount of time playing with war toys [20, 21] than are girls. Adolescent boys are exposed to more violent media, including violent video games and movies, than are girls [22]. As adults, men are more likely than are women to carry a deadly weapon [23], to engage in a physically aggressive altercation [24], and to be the victim of violence involving a weapon [25]. Patterns of sex differences in aggression are consistent cross-culturally [23, 26] and can be observed throughout history, at least as far back as Neolithic societies [27]. An attentional bias for weapons could therefore result from, or arise from the same mechanisms that produce gender-congruent object preferences and sex differences in the propensity to engage in violent behaviour.

Following violent game play, participants report more aggressive thoughts and are more likely to interpret an ambiguous scene as hostile [28]; exhibit enhanced interference by aggressive words in an emotional Stroop task [29]; and exhibit stronger associations between concepts of self and violence in an implicit association test [30]. The mere presence of a weapon (be it a word, an image or an actual weapon) even in children (especially boys, [31]), can promote aggressive behaviour, cognition, and affect [32, 33]. This is known as the weapons effect [34]. Men are more susceptible than are women to such primes [35–37].

The sex difference in the weapons effect mirrors that seen in attentional biases for weapons. Men also exhibit a stronger attentional bias for weapons than do women [9], and this effect is stronger when the weapons are depicted wielded [15]. While there is much evidence for sex differences in other cognitive domains [38–41], there remains only the two reports of sex differences in attentional bias for weapons. As such we know little about the functional implications of this sex difference men’s and women’s responses to weapons. One way to investigate these functional consequences is to examine how these biases manifest in the context of recent weapons-based violence.

In the present study, we used video games to create either a violent or non-violent context in which participants were then required to complete a visual search task, which measured their attentional bias for weapons (guns and weapon knives) compared to other non-weapon targets (staplers and knives used for cooking). Consideration of the weapons effect predicts a strengthening of the weapons bias following the violent video game, which would be consistent with the short-term increases in aggressive behaviour, cognition, and affect, that violent video game play is known to have [42–45]. Since men may be more prone to the weapons effect than are women [46], we also predicted that any effects of the violent video game would be more pronounced in men than in women.

Additionally, we attempted to investigate how cautiously participants behaved while they were searching for weapon targets. Caution is operationalized by comparing response times on target absent trials, with those on target present trials. Based on the notion that search on target-absent trials is terminated once a certain amount of time has passed without a target having been detected [6, 47], relatively long response times on target absent trials indicate increased caution–the participant is trading off response speed to reduce the likelihood of failing to detect a target. Participants exhibit increased caution to venomous (compared to non-venomous) animals, and a further increase in caution when venomous animals are depicted in peri-personal space [6]. They also exhibit increased caution when searching for weapons compared to non-weapons, especially so when the weapons are depicted wielded [9]. This suggests that the rapid decision-making that occurs during visual search tasks is derived from the implied real-world costs of failing to detect the weapon targets, as they are depicted. Failing to detect a wielded gun would likely be more costly than failing to detect a stationery gun sitting on a bench, and so participants exhibit more caution when searching for the former than when searching for the latter [9, 15].

In the current study, participants were required to search for weapons that were depicted as ’inanimate’ (sitting still on a surface, with no human in the image) and ’animated’ (depicted being held in someone’s hand in their regular functional position). By depicting the weapons wielded, we increased the immediacy of the threat they presented. We predicted that animated weapons would be searched for with greater caution than inanimate weapons or non-weapon targets, as weapons depicted wielded, as opposed to sitting passively on a surface, present a more immediate threat [9, 15]. It was also hypothesized that caution during search for weapons would increase following the violent video game (compared to following the non-violent game), since the real-world costs of failing to detect a weapon in a violent situation would be greater than those when in a non-violent situation. The primary novel hypotheses of the current study pertained to the impacts of violent video game play on both caution and response times, and we were primarily interested in any sex differences in these effects. To the extent that men are more prone to the impacts of violent video game play (being more prone to the weapons effect, [46]), we expected men, compared to women, to exhibit larger changes in response time and caution when searching for weapons after playing the violent, compared to the non-violent video game.

## Methods

The Charles Sturt University Human Research Ethics Committee approved this project. Participants provided written informed consent under protocol number 2015/064.

### Participants

We recruited 58 participants to both sessions of this study (29 women aged 18 to 59, *M* = 25.52, *SD* = 10.34; and 29 men aged between 18 to 55, *M* = 26.24, *SD* = 8.64), from an undergraduate participant pool (*N* = 8) and volunteers from the general public (*N* = 50). Three additional participants (2 men and 1 woman) did not return to complete their second session and their data were excluded from all analyses. An *a priori* power analysis for the within-between interaction in a repeated measures ANOVA, assuming an effect size of Cohen’s f = 0.15, η^2^_ρ_ = .02, correlations between measures of 0.5, adopting an alpha of 0.05, and requiring power of 0.9, suggests a required sample size of 52.

### Design

The experiment employed a mixed design, with threat (2 levels: weapon vs. non-weapon), context (2 levels: violent vs. non-violent video game), and animation (2 levels: items depicted wielded vs. un-wielded) as within-subjects variables and sex (2 levels: male vs. female), as a between-subjects variable.

Analyses also included a target group variable (guns vs. knives), which grouped each weapon with its control object (guns with staplers and weapon knives with cooking knives, respectively), and accounted for variance in responses between these groups. Distractor sets were yoked between grouped targets (e.g., the same distractor sets when searching for guns or staplers), but different sets were used between the target groups (i.e. different distractor sets when searching for guns as opposed to knives). This allowed robust comparisons between weapons and non-weapons and reduced repetition of distractors, keeping all visual displays as fresh as possible.

### Stimuli

#### Prime stimuli

Two video games, one violent and one non-violent, were chosen to prime participants. The violent video game was called Sin666 and is freely accessible online (URL: http://666games.net/Violent/Flash/Play/252/fullscreen.php). The premise of the game requires participants to shoot 30 people in order to join a gang. Participants are required to visually scan the screen for rivals and the game involves reflexive action to shoot using a gun. Players use a mouse to aim and click to shoot or reload. The non-violent videogame was called ’Capture the Dinosaur’, and was also freely available online, although it has since become unavailable (its prior URL: http://www.ogigames.com/play/5329/mega-rig-dinosaur-rescue-game). This game required players to ’chase’ a dinosaur: players must visually monitor the screen for obstacles and use reflexive action to dodge obstacles. Players also used a mouse to play this game. Thus both games adopted similar game play mechanisms. Participants played the requisite game for that session for 15 minutes immediately prior to commencing the visual search task.

#### Visual search stimuli

Stimuli (targets and distractors) were RGB colour photographs (converted to rectangles of 198x283 pixels at a resolution of 72 ppi using Adobe Photoshop v11.0.2 for Mac), and sourced from Google Image searches (and licensed for ’re-use and modification’, Creative Commons) and the private collection of the third author. Each trial of the visual search task presented a 3 x 3 array of nine such images (either 9 distractors in target-absent trials, or 8 distractors and 1 target in target-present trials) against a black background.

#### Visual search target stimuli

Targets were weapons (guns and weapon knives) and non-weapons (staplers and cooking knives) and were depicted ’animated’ (wielded or held) or inanimate (un-wielded, depicted passively on a surface, with no hand in the image). Nine different images of each target type (i.e., wielded or un-wielded guns, staplers, weapon knives or cooking knives) were used, with each individual target image appearing in only a single trial for each participant. The nine images of each target type, occupied each of the 9 positions in the 3 x 3 arrays once.

#### Visual search distractor stimuli

Four sets of distractor images were compiled. Each set included 81 images, 9 from each of 9 object categories: clock/watch, hairbrush, paintbrushes, tools (e.g. wrench, hammer), mugs, bowls, sports equipment (e.g. tennis racquet), books and bottles. Two of the four sets were made up of images that all included hands holding or manipulating the objects and were used as the distractors for the wielded guns/staplers and weapon knives/cooking knives target groups, respectively. The other two sets included images of the objects with no hands present and were used as the distractors for the unwielded guns/staplers and knives, respectively.

#### Visual search arrays

**Fig 1:**
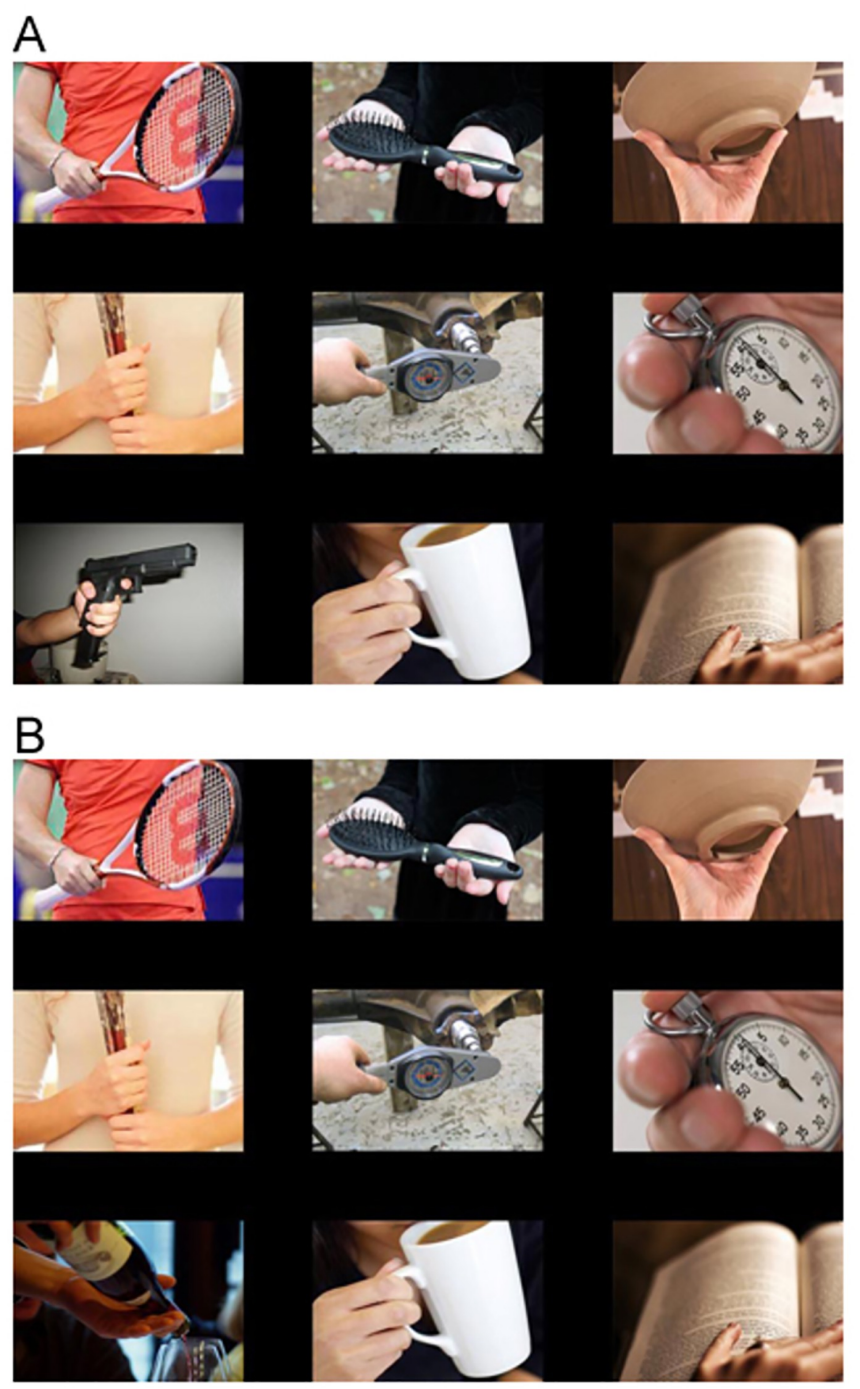
Shows two example arrays, a A) wielded gun target-present and B) corresponding target-absent trial.

Target-absent arrays contained one image from each of the nine distractor categories, with each distractor image appearing in just one target-absent array for each target type. Target-present arrays were then created by duplicating each target-absent array, and replacing one of the distractors with a target image. Using many different images within each condition, but identical arrays between target-absent and target-present trials both within a condition and between the to-be-compared target pairs (guns vs. staplers, for example) serves the dual purpose of ensuring that the arrays are visually complex and low in familiarity throughout the task, increasing external validity, and diminishing the likelihood that quirks of individual distractor images could drive any mean differences in responding between conditions [48]. Fig 1 illustrates example corresponding target present (Fig 1A) and target absent (Fig 1B) arrays for a gun target in the animated condition.

### Procedure

Participants were initially shown images of weapons (similar to those used in the study) and asked not to participate if they would be uncomfortable viewing the images, or if they had previously had a violent experience with weapons. No prospective participants withdrew from the study following these instructions.

Participants then played either the violent or non-violent video games for 15 minutes (a duration previously reported to elicit priming effects, Anderson et al., 2010). The order of video games played across the two sessions was counterbalanced across participants with half (15 women and 14 men) playing the violent video game in their first session.

Participants next completed the visual search task (controlled by Inquisit Version 4.3 Web, Millisecond). Trials were blocked by target type (i.e., wielded or un-wielded guns, staplers, weapon knives or cooking knives) with 18 trials (9 target-absent and 9 target-present) per block. Trials within blocks were presented in a randomized order for each participant. On screen instructions informed participants to search for either "guns", "staplers", or "knives" (used for both cooking and weapon knife conditions) at the beginning of each block and also informed participants to try to respond as quickly and as accurately as possible. Trials commenced with a fixation cross (for 500ms, including the name of the target below it), followed by the search array, which remained on the screen until participants indicated by key press whether the target was present or absent. The ’a’ and ’p’ keys were used to indicate absent and present, respectively. There was a 400ms inter-trial interval and no feedback regarding accuracy was given.

Following completion of the visual search tasks, participants provided their age and sex and indicated how frequently they played video games (never, less than once a week, once or twice a week, about every second day, most days, and every day) and what percentage of the games they play had violent content (none, less than half, about half, more than half, or all). Frequency of video game play was scored from 0 = never, to 5 = every day. Frequency of violent video game play was defined as the product of the frequency of video game play score, and the percentage of games played with violent content score (from 0 = none to 4 = all).

### Data analysis

For each target type we recorded each participant’s accuracy (proportion correct responses to target-present trials), reaction time (mean response time to target present trials, excluding individual response times longer than 10000ms and shorter than 250ms) and caution score. The caution score (C), is calculated as:

$$\text{C}=({\text{RT}}_{\text{absent}}-{\text{RT}}_{\text{present}})/({\text{RT}}_{\text{absent}}+{\text{RT}}_{\text{present}})$$

where RT_absent_ is the mean reaction to target-absent trials and RT_present_ is the mean reaction time to target-present trials. A higher caution score indicates that participants are waiting a comparatively longer time to declare that a target is absent, relative to the average time it takes them to locate the target when it is present. Caution scores are higher for more threatening targets [6, 9] and this is interpreted as implicitly attaching a higher cost to missing a potentially threatening target.

All analyses were conducted using IBM SPSS v27 for Mac. An alpha level of 0.05 was applied to all analyses. All raw data are provided within the supplementary materials.

## Results

To the accuracy and reaction time data we applied two mixed effects ANOVA models with threat (2 levels: weapons and non-weapons), context (2 levels: violent vs. non-violent video game), and target groups (2 levels: guns/staplers and knives) as within-subjects variables and with participant sex as a between-subjects variable. These models were applied to data from the wielded and unwielded conditions separately (following [9]), due to substantial mean differences expected between these respective conditions. Caution scores were subjected to a model which included all the above variables, plus another variable: animation (2 levels: wielded and unwielded), to accommodate all data into a single model (following [6, 9]), since caution scores are theoretically and empirically robust to large differences in response time attributable to factors such as display complexity (see [6] for a detailed account as to why this is the case). Two- and three-way interactions observed in these models were unpacked via planned contrasts and simple effects comparisons. For each dependent variable we offer a summary of key effects found, to help orientate the reader prior to reporting the full statistics for the models.

### Accuracy

Across both wielded and unwielded targets, weapons were located more accurately than were non-weapon objects, as expected. Men also located wielded weapons (but not non-weapon objects) more accurately than did women, but closer inspection of pairwise contrasts revealed that this difference was only significant for guns searched for only after playing the violent video game. Of less theoretical interest we also observed that guns/staplers were located more accurately than weapon/cooking knives. These results are reported in full below.

When targets were not depicted wielded, the expected main effect of threat was observed, (F(1,56) = 237.8, p < .001, η^2^_ρ_ = .809), as participants located weapons more accurately than they did non-weapon objects. There was also a main effect of target group, (F(1,56) = 69.4, p < .001, η^2^_ρ_ = .553), as guns and staplers were located more accurately than were knives, and a threat by target group interaction, (F(1,56) = 26.5, p < .001, η^2^_ρ_ = .322), as the simple effect of threat was larger between the knives (F(1,56) = 188.7, p < .001, η^2^_ρ_ = .771), than between the guns and staplers, (F(1,56) = 60.4, p < .001, η^2^_ρ_ = .519). No other main effects or interactions were significant (all other *p* > .248).

When targets were depicted wielded, the expected main effect of threat was again observed, (F(1,56) = 114.5, p < .001, η^2^_ρ_ = .672), as participants located weapons more accurately than did non-weapons. For these targets we also observed the predicted threat by sex interaction (F(1,56) = 4.817, p = .032, η^2^_ρ_ = .079), as men located weapons more accurately than did women (F(1,56) = 5.65, p = .021, η^2^_ρ_ = .092), with no sex difference observed for the non-weapon objects (F(1,56) = 0.56, p = .459, η^2^_ρ_ = .010). We also observed the same main effect of target group as was observed for the unwielded targets, (F(1,56) = 92.9, p < .001, η^2^_ρ_ = .624), as guns and staplers were located more accurately than were knives, and a threat by target group interaction, (F(1,56) = 27.7, p < .001, η^2^_ρ_ = .331), as the simple effect of threat was larger between the knives (F(1,56) = 123.9, p < .001, η^2^_ρ_ = .689), than between the guns and staplers, (F(1,56) = 33.4, p < .001, η^2^_ρ_ = .373).

**Fig 2:**
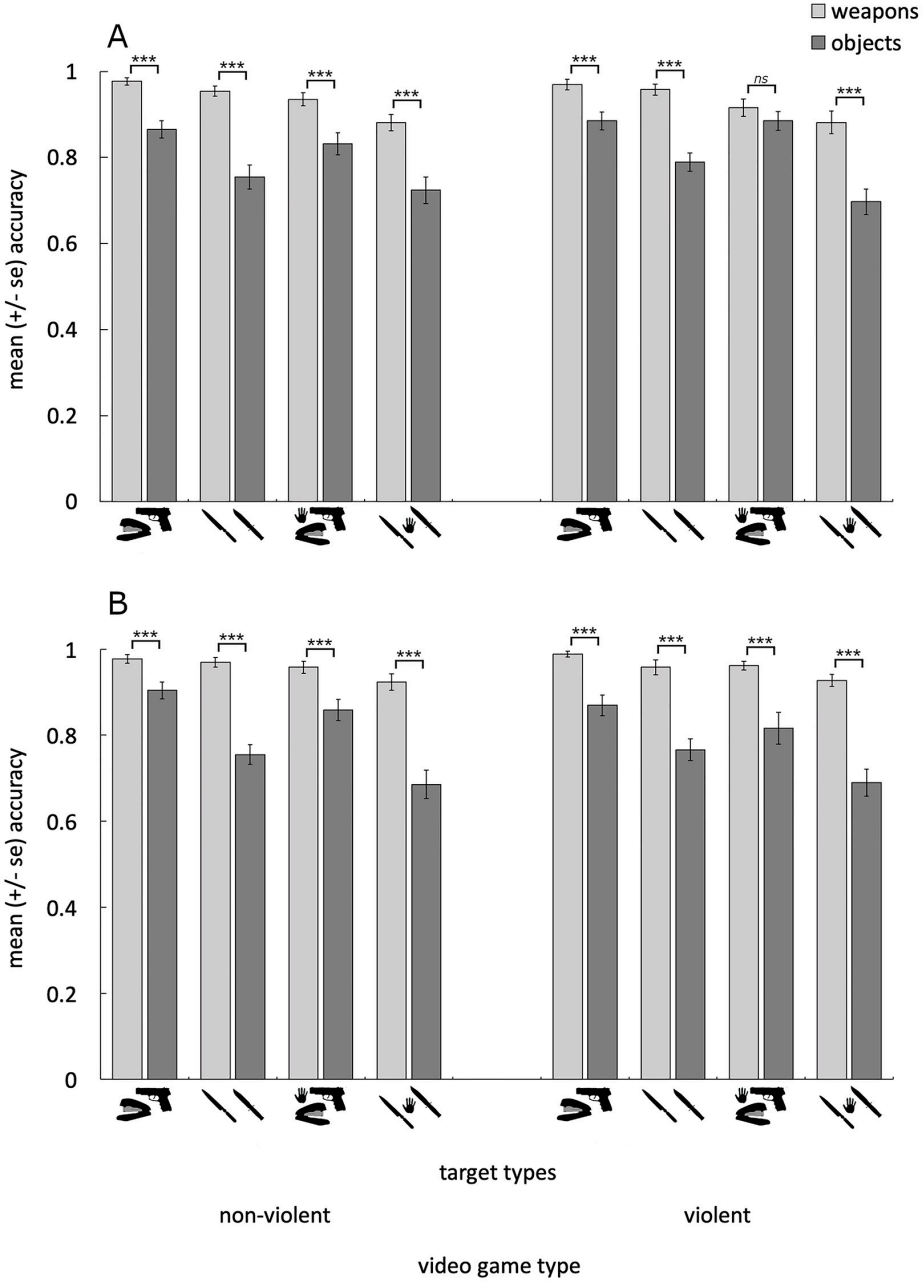
Shows the mean (±se) accuracy when locating the weapon and non-weapon targets for women (A) and men (B) after playing the non-violent (left) and violent (right) video games. Weapons were located more accurately overall than were non-weapon targets. ***p < .001, **p < .01, *ns* = not significant.

In addition, we also observed a four-way interaction (involving threat, target group, context, and sex), that approached significance (F(1,56) = 3.731, p = .058, η^2^_ρ_ = .062). Pairwise comparisons investigating the full-factorial simple effects of sex, revealed that men located only the guns more accurately than did women, and only after playing the violent video game (F(1,56) = 4.11, p = .048, η^2^_ρ_ = .068). No other simple effects of sex were significant (all p > .125). These results are depicted in Fig 2.

### Reaction time

As predicted, weapons were located more quickly than were non-weapon objects, whether depicted wielded or not. Also as predicted, men found both the wielded and unwielded weapons (but not the non-weapon objects) more quickly than did women. While men located guns more quickly than did women regardless of which video game they had just played, sex differences in searching for unwielded weapon knives (and also cooking knives) emerged only after playing the violent video game. The full statistical results from these models are reported below.

When targets were not depicted wielded, we observed the expected main effect of threat (F(1,56) = 176.0, p < .001, η^2^_ρ_ = .759), as weapons were located more quickly than were non-weapon objects. There was also a main effect of target group, (F(1,56) = 114.8, p < .001, η^2^_ρ_ = .672), as guns and staplers were located more quickly than were knives, and a threat by target group interaction, (F(1,56) = 11.5, p < .001, η^2^_ρ_ = .171), as the simple effect of threat was smaller between the knives (F(1,56) = 70.2, p < .001, η^2^_ρ_ = .556), than it was between the guns and staplers, (F(1,56) = 107.4, p < .001, η^2^_ρ_ = .657).

We also observed significant target group by threat by sex (F(1,56) = 4.784, p = .033, η^2^_ρ_ = .079) and context by target group (F(1,56) = 5.793, p = .019, η^2^_ρ_ = .094) interactions. Linear contrasts confirmed that men located the weapons (F(1,56) = 8.22, p = .006, η^2^_ρ_ = .128), but not the non-weapon objects (F(1,56) = 2.74, p = .103, η^2^_ρ_ = .047) more quickly than did women. Pairwise comparisons investigating the full-factorial simple effects of sex, revealed that men located the guns more quickly than did women after playing both the violent (F(1,56) = 10.3, p = .002, η^2^_ρ_ = .155) and non-violent (F(1,56) = 8.05, p = .006, η^2^_ρ_ = .126) video games, but only located the knives (both weapon and cooking) more quickly after the violent video game (weapon: F(1,56) = 6.91, p = .011, η^2^_ρ_ = .110; cooking: F(1,56) = 5.98, p = .018, η^2^_ρ_ = .097). No other simple effects of sex were significant (all p > .136).

When the targets were depicted wielded, we again observed the predicted effect of threat F(1,56) = 133.9, p < .001, η^2^_ρ_ = .705), as weapons were located more quickly than were non-weapon objects. There was also a main effect of target group, (F(1,56) = 80.62, p < .001, η^2^_ρ_ = .590), as guns and staplers were located more quickly than were knives, and a threat by target group interaction, (F(1,56) = 23.16, p < .001, η^2^_ρ_ = .293), as the simple effect of threat was smaller between the two types of knives (F(1,56) = 21.5, p < .001, η^2^_ρ_ = .278), than between the guns and staplers, (F(1,56) = 132.9, p < .001, η^2^_ρ_ = .704).

**Fig 3:**
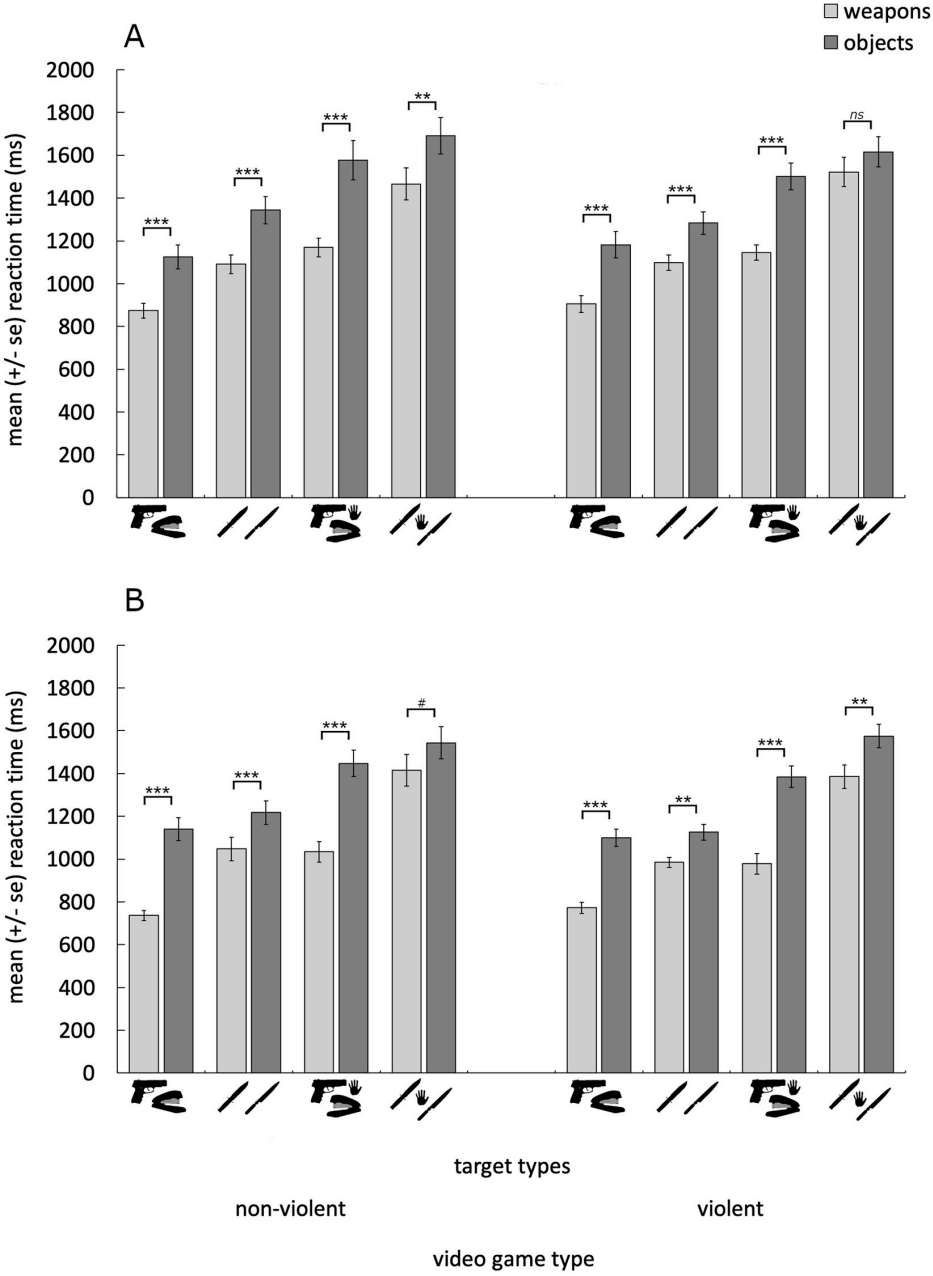
Shows the mean (±se) response time to locate the weapon and non-weapon targets for women (A) and men (B) after playing the non-violent (left) and violent (right) video games. Weapons were located more quickly overall, than were non-weapon targets. ***p < .001, **p < .01, #*p*<0.1, *ns* = not significant.

We also observed a significant main effect of sex (F(1,56) = 4.088, p = .048, η^2^_ρ_ = .068). Although no interactions involving either sex or context were significant (all p > .165) planned linear contrasts revealed that men found only the weapons (F(1,56) = 4.20, p = .045, η^2^_ρ_ = .070) and not the non-weapon objects (F(1,56) = 2.87, p = .096, η^2^_ρ_ = .049) significantly more quickly than did women. Pairwise comparisons investigating the full-factorial simple effects of sex, revealed that men located the guns more quickly than did women after playing both the violent (F(1,56) = 7.77, p = .007, η^2^_ρ_ = .122) and non-violent (F(1,56) = 4.33, p = .042, η^2^_ρ_ = .072) video games, with no other significant simple effects of sex (all p > .128). These results are depicted in Fig 3.

### Caution

As expected, participants exhibited more caution when searching weapons compared to when searching for non-weapon targets. Women exhibited more caution when searching unwielded weapons (compared to unwielded objects) after playing the violent video game (but not after playing the non-violent game). Men similarly exhibited more caution when searching for wielded weapons (compared to wielded objects) after playing the violent video game (but not after playing the non-violent video game). Contrary to expectations, however, these effects arose not because of increases in caution exhibited when searching for weapons after the violent video game, but because of significantly decreased caution exhibited when searching for non-weapon objects after the violent video game. Also contrary to expectations, participants exhibited less overall caution when searching wielded (compared to unwielded) weapons. These results are reported in full below.

In the model combining data from the wielded and not wielded conditions, we observed a significant main effect of threat (F(1,56) = 45.87, p < .001, η^2^_ρ_ = .450) as more caution was exhibited when searching for weapons than when searching for non-weapon objects. There was also a significant main effect of target group (F(1,56) = 81.0, p < .001, η^2^_ρ_ = .591), as participants also exhibited more caution when searching for guns / staplers than when searching for knives. A significant main effect of animation was also observed (F(1,56) = 12.84, p < .001, η^2^_ρ_ = .187), but contrary to expectations participants actually exhibited less caution when searching for wielded guns (F(1,56) = 5.25, p = .026, η^2^_ρ_ = .086) and weapon knives (F(1,56) = 6.60, p = .013, η^2^_ρ_ = .105), though not wielded staplers (F(1,56) = 2.64, p = .110, η^2^_ρ_ = .045) and cooking knives (F(1,56) = 2.72, p = .105, η^2^_ρ_ = .046), compared to their unwielded counterparts.

The main effects of threat and target group, were also qualified by a significant three-way interaction between threat, target group, and sex (F(1,56) = 5.820, p = .019, η^2^_ρ_ = .094). The three-way interaction is accounted for as (averaged across context and animation) men exhibited more caution than did women when searching for guns (F(1,56) = 5.60, p = .021, η^2^_ρ_ = .091), but not when searching for staplers (F(1,56) = 0.46, p = .501, η^2^_ρ_ = .008), weapon knives (F(1,56) = 0.05, p = .827, η^2^_ρ_ = .001), or cooking knives (F(1,56) = 3.92, p = .053, η^2^_ρ_ = .065).

And lastly, we observed a significant context by animation by threat by sex four-way interaction (F(1,56) = 13.455, p < .001, η^2^_ρ_ = .194). Simple effects contrasts revealed that the most parsimonious explanation for the four-way interaction was the emergence of simple effects of threat after playing the violent video game, that were not apparent after playing the non-violent video game. Women did not exhibit a significant simple effect of threat for unwielded weapons after playing the non-violent game (F(1,56) = 0.11, p = .745, η^2^_ρ_ = .002), but did so after playing the violent video game (F(1,56) = 25.26, p < .001, η^2^_ρ_ = .311). Intuitively, it may be expected that this effect would have occurred as a result of women increasing the caution they displayed when searching for unwielded weapons after playing the violent video game. But this was not the case (F(1,56) = 0.99, p = .323, η^2^_ρ_ = .017). Rather, women significantly decreased the caution they exhibited when searching for non-weapon objects after playing the violent (compared to the non-violent) video game (F(1,56) = 7.06, p = .010, η^2^_ρ_ = .112)

**Fig 4:**
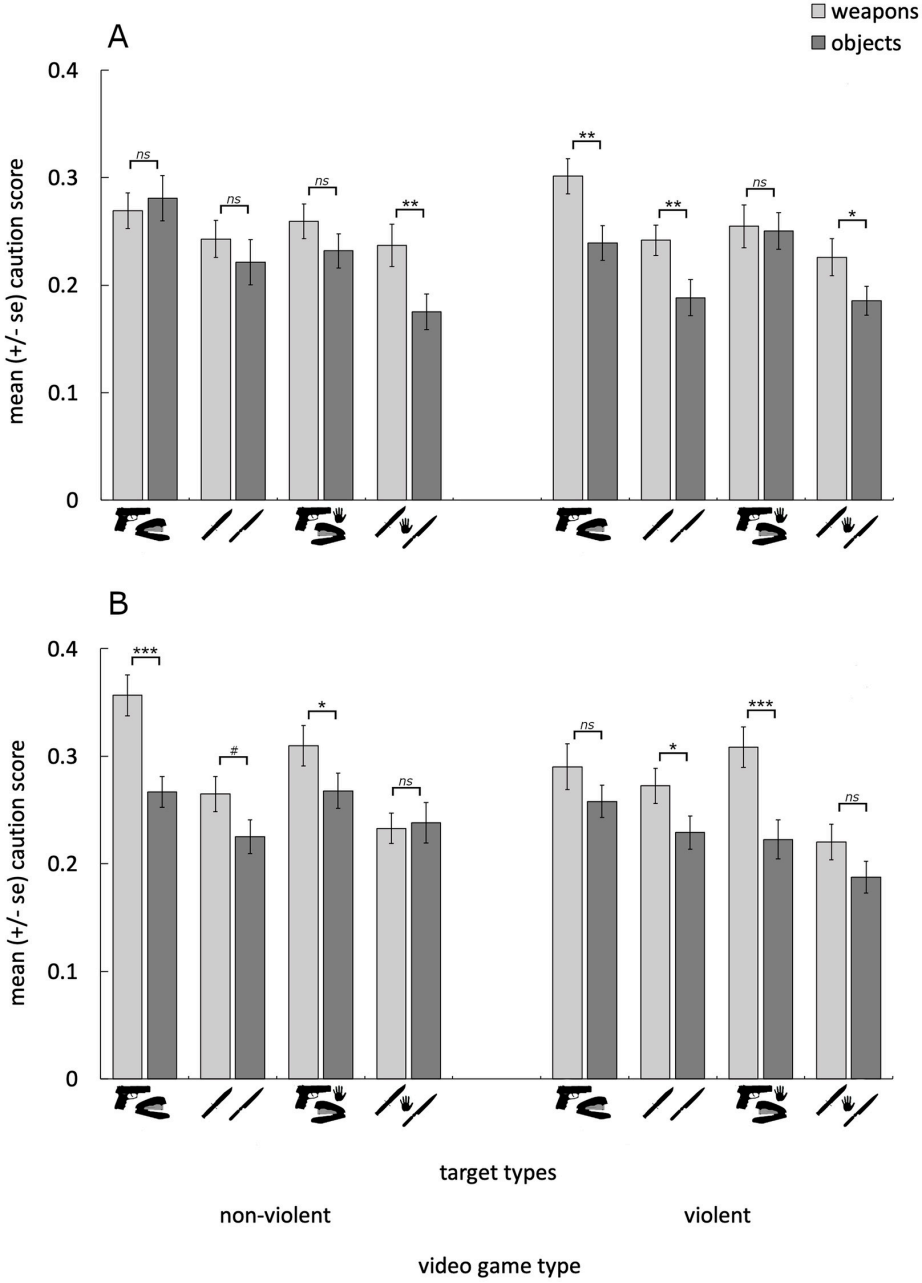
Shows the mean (±se) caution exhibited when locating the weapon and non-weapon targets for women (A) and men (B) after playing the non-violent (left) and violent (right) video games. Women exhibited simple effects of threat for unwielded weapons after playing the violent, but not the non-violent, video game, while men exhibited stronger simple effects of threat for wielded weapons after violent (compared to the non-violent) video game. In both cases, these effects were attributable to reduced caution exhibited toward the non-weapon objects, after playing the violent (compared to the non-violent) video game. ***p < .001, **p < .01, *p < .05, #*p*<0.1, *ns* = not significant.

Similarly, men did not exhibit a significant simple effect of threat for the wielded weapons after the non-violent video game (F(1,56) = 1.67, p = .201, η^2^_ρ_ = .029), but did so after the violent video game (F(1,56) = 16.61, p < .001, η^2^_ρ_ = .229). And as above, this was attributable not to an increase in caution when searching for weapons (F(1,56) = 0.75, p = .390, η^2^_ρ_ = .013), but to a decrease in caution when searching for non-weapons (F(1,56) = 8.21, p = .006, η^2^_ρ_ = .128) after playing the violent video game. These results are illustrated in Fig 4.

### Effects of real-world video game experience

Since men engaged in real-world video game play (total: t(32.3) = 5.40, p < .001; and violent: t(30.3) = 4.98, p < .001) substantially more than did women, frequencies of total and violent video game play could not be entered as covariates into the above models (due to violations of the covariate assumption of equal means between groups). Instead, for each of three dependent variables, 2x2x2x2 within-subjects models (with threat, context, animation, and target group as variables) were applied to male and female data separately, with frequency of all video game play and violent video game play entered as (centred) covariates, respectively.

For women, the frequency of all video game play significantly predicted response times (F(1,27) = 4.379, p = .046, η^2^_ρ_ = .140), as those who played video games more often tended to respond more quickly. For male participants, frequency of violent video game play tended to predict response times (F(1,27) = 3.017, p = .094, η^2^_ρ_ = .101), as men who played violent video games more often tended to respond more quickly. No other covariate main effects were significant, all p > .206, all η^2^_ρ_ < .059.

Significant interaction terms between the covariates and within-subjects variables in the models would suggest that frequency of real-world video game play moderates some of the main effects and interactions previously reported. With three dependent variables, and separate models applied to the male and female data, however, there were a total of 90 such possible interactions to inspect. This created substantial likelihood of Type I errors, with 2–3 false positive interactions expected for each sex. For women, three significant interaction terms were observed, approximating the number of false positives expected. For men, six significant interaction terms were observed, somewhat exceeding the number of false positives expected. (S1 File) describes these interactions and the extent to which they could provide evidence for a moderating role for real-world video game play in the effects reported. This potential moderating role is not considered further in the main manuscript as the statistical evidence for it is equivocal at best.

## Discussion

In this study we examined the impact of violent video game play on sex differences in attention biases for weapons. Broadly speaking, we replicated the threat superiority effect, observing that weapons were generally located more accurately, more quickly, and with more caution than were non-weapon targets. This re-affirms the threat superiority effect for weapons as a robust phenomenon, even when the non-threatening targets are superficially similar in shape and appearance to the weapons, a criticism of some previous reports [48]. We also confirmed previous reports of sex differences in search behaviour for weapons [9, 15] with men locating weapons more quickly than did women, and guns in particular, more cautiously. This confirms that both attention bias and rapid decision-making processes with respect to weapons robustly differ between men and women.

Violent video game play impacted participant responses across the three dependent measures. With respect to accuracy and reaction time, sex differences emerged after playing the violent video game, that were not apparent after playing the non-violent video game. Men located the wielded guns more accurately than did women only after playing the violent video game. Men also located both weapon knives and cooking knives more quickly than did women, only after playing the violent video game. These findings suggest that, as well as men tending to exhibit stronger spontaneous attention biases for weapons [9] especially when depicted wielded [14], these biases may also be more responsive to violent contexts in men than they are in women. This would be consistent with prior observations that men may be more prone to the weapons effect than are women [46], see also [49]. Importantly, though, the detectable effects of the violent game were modest. We did not observe an across-the-board increase in speed and accuracy sex differences after playing the violent video game, which might have been expected if men’s responses to weapons were substantially more receptive to violent contexts than are women’s responses.

The impact of violent video game play on the level of caution exhibited during search was similar in men and women, and somewhat counterintuitive. In both sexes simple effects of threat were observed (with more caution exhibited when searching for weapons than when searching for non-weapon objects) after playing the violent video game, that were not observed after playing the non-violent video game. For women, this occurred with respect to the unwielded targets, while for men this occurred with respect to the wielded targets. Surprisingly, however, for both sexes these effects were not due to greater caution exhibited when searching for weapons after watching the violent video game. Rather, they were attributable to a significant decrease in exhibited caution when searching for non-weapon objects, after playing the violent, compared to the non-violent, video game.

These findings are not immediately consistent with the proposition that a threatening context increases the implicit cost of failing to locate a weapon, thus increasing the caution with which weapons are searched for. These findings could be accounted for, however, by supposing that participants were not just attempting to find each target as quickly as possible (within each trial), but (after playing the violent video game) were attempting to find *all* weapons targets, across trials, as quickly as possible. In doing so, participants may have been willing to increase their odds of missing non-weapon targets, by hurrying responses in those conditions, thus resulting in the lower caution scores, and faster overall completion of those conditions.

The explanation here relies on the possibility that participant decision-making in this task is not just about the speed-accuracy trade-off within each trial. Rather, speed-accuracy trade-offs across different conditions could be occurring. We are supposing that after playing the violent video game, (but not after playing the non-violent game), participants were motivated to complete the blocks of trials in which they were searching for a non-weapon as quickly as possible (creating fast target-absent responses and low caution scores), so that they could resume their search for a weapon as quickly as possible. Under this explanation, caution when searching for weapons is maintained after playing the violent video game. This is because it is the motivation to locate weapons as soon and as accurately as possible, that drives the fast and careless responding when searching for non-weapon objects. Although a *post hoc* explanation, it is interesting to consider that such cross-condition effects could occur in tasks for which it is typically assumed that performance on respective trials, and blocks, is largely independent.

To test whether this explanation is correct, we would need to design a study which varies both the context (including violent and non-violent video game primes as the current study did) and the specific set of targets to be located. Some participants would search for both weapons and non-weapon targets, other participants would search for non-weapon targets only. If the above explanation for the reduction in caution exhibited toward non-weapon targets after the violent video game is correct, then reductions in caution should not be observed in violent contexts (compared to benign contexts) when the only targets to be searched for across the whole experiment are non-weapon objects. This is because participants in such a study would not be expecting to ever be offered an opportunity to search for weapons. They would therefore have no motivation to rush through the non-weapon target trials in order to sooner resume their search for weapons. In fact, in the violent context (relative to the non-violent context), participants may even exhibit more caution when searching for non-weapons objects, since even non-weapons can be weaponized in an emergency. Only when there is an expectation that weapons targets will also be presented (because the participant is aware that the experiment involves searching for both weapons and non-weapons), would we predict less caution to be exhibited when searching for non-weapons targets. A future study designed in just such a way would be able to verify whether our interpretation here is correct.

Neither sex exhibited any evidence of increased caution when searching for animated, compared to inanimate weapons, but actually exhibited decreased caution for animated non-weapons and weapons in some circumstances. This is an unexpected finding that is not consistent with previous reports that both sexes exhibited more caution when searching for animated, compared to inanimate weapons, with no such corresponding effect for non-weapon targets [9, 15]. Increases in caution when searching for wielded weapons (compared to unwielded weapons), therefore, may not be a robust phenomenon. Some unknown factor leading to an effect of animation on caution when searching for weapons may have been present in the prior studies or their samples, but not in the current study. Given the similarity in sample characteristics and details of the respective studies’ designs, however, this does not seem a likely explanation.

Alternatively, the video game primes may have impacted participants’ response strategies in such a way as to systematically reduce caution for longer response time categories. This could occur if participants under-estimated their own target-present response times in a non-linear way (larger underestimates for longer response times). The amount of caution exhibited is presumed to reflect participants’ perceptions of their mean target-present response time, interacting with their decision-making processes on target-absent trials. On target-absent trials, participants perceive the passage of time. As this exceeds their mean target-present response time, it becomes increasingly likely that were this a target-present trial, the participant would have spotted the target by now. As such, the more time passes on a target-absent trial, the more likely the participant becomes to declare the target absent. Exactly how much past their average target-present response time the participant waits to make this decision, reflects the level of caution they are exhibiting. The longer they wait, the more time they are willing to invest in minimizing the chances that they will miss a target, that is actually there. Prior studies reporting increased caution when searching for wielded, compared to unwielded, weapons have not included any primes. If the presence of the primes somehow caused participants to systematically underestimate their own target-present response times, this would produce lower caution scores. If this underestimation was greater for longer response time trials, this would account for why less caution was observed when searching for wielded, compared to unwielded weapons in the current study: absolute response times are always substantially longer in ‘held’ compared to ‘unheld’ conditions in these paradigms, due to increased target-distractor similarity [6, 9, 15]. The problem with this explanation is that a coherent mechanistic explanation as to how the presence of the primes could interfere with participants’ perception of their own target-present response times in this particular way, does not immediately present itself.

The caution score may, however, be prone to such vagaries. As it is currently calculated, the caution score compares the relative mean response times in target absent and target present conditions, on the assumption that participants quickly learn how long on average a target in a particular condition takes to find, and then waits some portion of time longer than this on absent trials, prior to responding. It does not consider that some target-absent responses will occur prior to the participant having an opportunity to learn what a ’typical’ target-present response is. Perhaps more importantly, it does not consider variance in target present response times across trials. Consider a participant with a target-present response time distribution with a mean of 1000ms, and a standard deviation of 100ms. If this participant waited 1300ms before responding on target absent trials, this would be three standard deviations longer than their mean target-present response time, and could be considered quite cautious responding. Another participant may have a target-present response time distribution with a mean of 1000ms, but a standard deviation of 150ms. In this case, waiting 1300ms before responding only corresponds to two standard deviations above their mean target-present response time. This participant is arguably responding less cautiously, but the current caution score does not capture this behavioural difference. The number of trials (9 target absent and 9 target present) in each condition of this study precludes the kind of modelling that would be needed to compare alternative formulas for calculating caution. Such applications would demand more than an order of magnitude increase in the number of trials [for example, 50], far exceeding the number of trials ever used in these types of threat-superiority, visual search tasks. Future studies could, however, investigate the distribution of the caution score, and whether mean response times on target-absent trials are indeed influenced by the variance of target-present response times, as well as their mean. Caution scores in prior studies have not shown susceptibility to these issues [6, 9, 14, 51], and so an explanation for why this may be a problem in the current study and not previous studies remain elusive. But this is still a possibility that should be investigated. Future studies should systematically interrogate how the caution score behaves when participants are induced to produce a wide range of response time means and variances, under varying task demands.

## Conclusions

In the current study we investigated the impact of a violent context, induced by briefly playing a violent video game, on attentional biases for weapons in men and women exhibited during a visual search task. We replicated the threat superiority effect for weapons and confirmed previously reported sex differences (favouring men) in attentional biases for guns. We also observed more sex differences in the visual search task’s behavioural measures after participants had played the violent video game, than after they had played the non-violent game. These results are consistent with the weapons effect (an increase in violence-related attitudes and cognitions after exposure to weapons) impacting visual attention. They also suggest that men were more susceptible than were women to the violent video game’s impact on visual search task performance. Observed sex differences after playing the violent video game were far from consistent across all weapons targets, however, suggesting that sex differences in the impacts of violent video-game play on visual attention for weapons are really quite modest. We also observed unexpected impacts of the violent video game on men’s and women’s decision-making biases when searching for non-weapons targets. We attribute these unexpected effects to participants de-valuing the non-weapon targets after playing the violent video game. We proposed that participants were willing to increase the risk of missing such targets in order to complete those trials faster, so that they could return more quickly to the more situationally adaptive task of searching for weapons. Future studies, however, are needed to verify this explanation. Lastly, we suggest that future studies systematically interrogate the caution score, to establish whether the variance as well as the mean of target-present response times, dictate the timing of decisions on target-absent trials.

## Supporting information

S1 File Analyses of real-life video game experience as moderators of performance effects in the visual search task. (DOCX)

S2 File (XLSX)
