# Supplementary material for: Using video games to understand sex differences in attentional biases for weapons
Source: PLoS One. 2022 Dec 22;17(12):e0279360. doi: 10.1371/journal.pone.0279360 (PMC9778952; doi:10.1371/journal.pone.0279360)
Supplement: S1 File — (DOCX) [file pone.0279360.s001.docx]

**Using Video Games to Understand Sex Differences in Attentional Biases for Weapons**

**Authors:**

Gemma van Heyst^1^

Myoungju Shin^1^

Danielle Sulikowski^1,2^

^1^School of Psychology, Charles Sturt University, Bathurst, Australia

**Supplementary Material:**

S1. Analyses of real-life video game experience as moderators of performance effects in the visual search task

**S1. Analyses of real-life video game experience as moderators of performance effects in the visual search task**

Since men engaged in real-world video game play (total: *t*(32.3)=5.40, *p*<.001; and violent: *t*(30.3)=4.98, *p*<.001) substantially more than did women, frequencies of total and violent video game play could not be entered as covariates into the above models (due to violations of the covariate assumption of equal means between groups). Instead, for each of three dependent variables, 2x2x2x2 within-subjects models (with threat, context, animation, and target group as variables) were applied to male and female data separately, with frequency of all video game play and violent video game play entered as (centred) covariates, respectively.

For women, the frequency of all video game play significantly predicted response times (*F*(1,27)=4.379, *p*=.046, *η^2^_ρ_*=.140), as those who played video games more often tended to respond more quickly. For male participants, frequency of violent video game play tended to predict response times (*F*(1,27)=3.017, *p*=.094, *η^2^_ρ_*=.101), as men who played violent video games more often tended to respond more quickly. No other covariate main effects were significant, all *p*>.206, all *η^2^_ρ_*<.059.

Significant interaction terms between the covariates and within-subjects variables in the models would suggest that frequency of real-world video game play moderates some of the main effects and interactions previously reported. With three dependent variables, and separate models applied to the male and female data, however, there were a total of 90 such possible interactions to inspect. This created substantial likelihood of Type I errors, with 2-3 false positive interactions expected for each sex.

For women, three significant interaction terms were observed, approximating the number of false positives expected. These all occurred in the models applied to the caution scores. When the frequency of all video game play was included as a covariate, a significant three-way threat by animation by all video game play interaction was observed (*F*(1,27)=4.795, *p*=.037, *η^2^_ρ_*=.151). This interaction was interrogated by estimating the two-way threat by animation interaction at low and high levels of the covariate (two standard deviations above and below the mean, respectively). At low levels of all video game play the two-way threat by animation interaction was marginally significant (*F*(1,27)=4.165, *p*=.051, *η^2^_ρ_*=.134), as participants exhibited greater caution when searching for wielded weapons, compared to non-weapon objects (*p*=.005, *η^2^_ρ_*=.256), with no simple effect of threat for unwielded weapons and objects (*p*=.643, *η^2^_ρ_*=.008). At high levels of all video game play, the two-way threat by animation interaction was not significant, (*F*(1,27)=3.571, *p*=.070, *η^2^_ρ_*=.117) and no simple effects of threat were observed when targets were depicted held (*p*=.915, *η^2^_ρ_*<.001), or not held (*p*=.061, *η^2^_ρ_*=.124).

When frequency of violent video game play was included as a covariate, two significant three-way interactions were observed: a threat by animation by violent video game play interaction (*F*(1, 27)=4.606, *p*=.041, *η^2^_ρ_*=.146); and a target type by animation by violent video game play interaction (*F*(1, 27)=5.084, *p*=.032, *η^2^_ρ_*=.158). The same analysis strategy as described above was applied to understand each of these three-way interactions. At low levels of violent video game play the two-way threat by animation interaction was marginally significant (*F*(1,27)=4.007, *p*=.055, *η^2^_ρ_*=.129), as participants exhibited greater caution when searching for wielded weapons, compared to non-weapon objects (*p*=.030, *η^2^_ρ_*=.162), with no simple effect of threat for unwielded weapons and objects (*p*=.928, *η^2^_ρ_*<.001). At high levels of violent video game play, the two-way threat by animation interaction was not significant, (*F*(1,27)=3.425, *p*=.075, *η^2^_ρ_*=.113). No simple effect of threat was observed when targets were depicted held (*p*=.591, *η^2^_ρ_*=.011), however, significantly more caution was exhibited towards inanimate weapons, compared to non-weapon objects (*p*=.015, *η^2^_ρ_*=.198).

Considering the second three-way interaction observed (target type by animation by violent video game play), at low levels of violent video game play the two-way target type by animation interaction was significant (*F*(1,27)=4.880, *p*=.036, *η^2^_ρ_*=.153), as participants exhibited more caution when searching for inanimate guns/staplers compared to knives (*p*<.001, *η^2^_ρ_*=.407), with no simple effect of target type when targets were depicted held (*p*=.364, *η^2^_ρ_*=.031). At high levels of violent video game play, the two-way target type by animation interaction was not significant (*F*(1,27)=3.380, *p*=.077, *η^2^_ρ_*=.111), but simple effects of target type were somewhat reversed compared to what was observed at low levels of violent game play. Greater caution was exhibited towards animate guns/staplers compared to animate knives (*p*=.023, *η^2^_ρ_*=.178), but no simple effects of target type were observed for targets depicted held (*p*=.836, *η^2^_ρ_*=.002).

In summary, then, for female participants, more overall real life video game experience tended to attenuate the simple effects of threat observed for the caution score, while real life experience with violent video games, tended to attenuate caution expressed toward wielded weapons, while increasing caution expressed towards unwielded weapons. No theoretical explanation that would bring cohesion to these three interaction terms immediately presents itself. Further, given that the number of significant interaction terms observed across all DVs approximated the number of false positives expected (given the Type I error rate given by an alpha of 0.05), it is difficult to conclude that the above effects constitute anything more than Type I errors, within the female sub-sample.

For men, six significant interaction terms were observed, somewhat exceeding the number of false positives expected. These were observed across the accuracy (4) and reaction time (2) dependent variables. When the frequency of all video game play was included as a covariate in the accuracy model, a three-way context by animation by frequency of game play was observed (*F*(1,27)=6.334, *p*=.018, *η^2^_ρ_*=.190), qualified by a four-way context by animation by threat by frequency of game play (*F*(1,27)=7.757, *p*=.010, *η^2^_ρ_*=.223). Both these interactions were interrogated by examining the three-way context by animation by threat interaction at low and high levels of video game play, respectively. At low levels of video game play this interaction was significant (*F*(1,27)=5.364, *p*=.028, *η^2^_ρ_*=.166), and accounted for by the fact that men were more accurate when searching for unwielded weapons (compared to objects, *p*<.001, *η^2^_ρ_*=.455, with no simple effect of threat for wielded targets, *p*=.070, *η^2^_ρ_*=.117) after playing the violent game. After playing the non-violent video game, though, the simple effect of threat was present for wielded (*p*=.033, *η^2^_ρ_*=.158), though not the unwielded (*p*=.149, *η^2^_ρ_*=.076) targets. At high levels of video game play, the three-way context by animation by threat interaction was also significant (*F*(1,27)=7.201, *p*=.012, *η^2^_ρ_*=.211), although the subsequent simple effects comparisons revealed a different pattern. In this case, men located both wielded and unwielded the weapons more accurately than their respective non-weapon targets after playing both types of video game (all *p*<.023, all *η^2^_ρ_*=.177). Therefore, real life experience with video game play increased the threat superiority effects exhibited by men, with respect to accuracy.

When frequency of violent video game play was added to the accuracy models as a covariate, we again observed a significant three-way threat by animation by video game play frequency interaction (*F*(1,27)=4.403, *p*=.045, *η^2^_ρ_*=.140), qualified by a significant four-way context by threat by animation by video game play interaction (*F*(1,27)=4.669, *p*=.040, *η^2^_ρ_*=.147). Further exploration of the three-way context by threat by animation interaction across low and high levels of violent video game play revealed that at low levels of game play the three-way interaction was not significant (*F*(1,27)=3.112, *p*=.089, *η^2^_ρ_*=.103), but men located wielded weapons less accurately than unwielded weapons after playing both the violent (*p*=.002, *η^2^_ρ_*=.292) and non-violent (*p*=.011, *η^2^_ρ_*=.219) video game (with no simple effects of animation for non-weapon objects, both *p*>.068, both *η^2^_ρ_*<.118). At high levels of violent video game play, however the three-way interaction was significant (*F*(1,27)=4.472, *p*=.004, *η^2^_ρ_*=.142), as men located objects depicted wielded less accurately than when depicted unwielded, only after playing the violent video game (*p*=.023, *η^2^_ρ_*=.410), with no other simple effects of animation present (all *p*>.410, both *η^2^_ρ_*<.025). Therefore frequency of violent video game play impacted the patterns of animation on accuracy (tending impact weapons more at low levels of violent play, and non-weapon objects more at high levels of violent game play), although it isn’t immediately obvious that this finding has direct theoretical implications.

When frequency of all video game play was added to the reaction time model, as a covariate, two significant four-way interactions were observed: context by animation by threat by video game play (*F*(1,27)=6.121, *p*=.020, *η^2^_ρ_*=.185); and threat by target type by context by video game play (*F*(1,27)=5.388, *p*=.028, *η^2^_ρ_*=.166). These interactions were each further explored independently.

At low levels of video game play, the three-way context by animation by threat interaction was significant (*F*(1,27)=7.429, *p*=.011, *η^2^_ρ_*=.216) as men exhibited significant simple effects of threat for both wielded (*p*=.002, *η^2^_ρ_*=.296) and unwielded (*p*=.011, *η^2^_ρ_*=.215) targets after playing the violent video game, but only after the unwielded (*p*<.001, *η^2^_ρ_*=.487), but not wielded (*p*=.305, *η^2^_ρ_*=.039) targets after playing the non-violent video game. At high levels of video game play, the three-way interaction was not significant (*F*(1,27)=2.943, *p*=.098, *η^2^_ρ_*=.098), as participants exhibited significant simple effects of threat across all targets and after playing both types of game (all *p*<0.28, all *η^2^_ρ_*>.167). Therefore, similar to the observations for the accuracy data, more real-world experience with video game play, tended to increase the threat superiority effects observed in the reaction time data.

Lastly, the three-way threat by target type by context interaction was not significant at either low (*F*(1,27)=0.171, *p*=.682, *η^2^_ρ_*=.006) or high (*F*(1,27)=0.037, *p*=.849, *η^2^_ρ_*=.001) levels of video game play. As such further explorations for the differential patterns across these variables as participants varied on frequency of real world video game play did not progress, as the initial four-way interaction likely resulted from a multitude of complementary contrasts that would be difficult, if not impossible, to identify.
